# Haematic antegrade repriming to enhance recovery after cardiac surgery from the perfusionist side

**DOI:** 10.1051/ject/2023004

**Published:** 2023-03-24

**Authors:** Juan Blanco-Morillo, Diego Salmerón Martínez, Jose M. Arribas-Leal, Piero Farina, Luc Puis, Angel J. Sornichero-Caballero, Sergio J. Cánovas-Lόpez

**Affiliations:** 1 Cardiac Surgery Department, Virgen de la Arrixaca University Hospital 30120 Murcia Spain; 2 Department of Health and Social Sciences, Murcia University 30120 Murcia Spain; 3 IMIB-Arrixaca 30120 Murcia Spain; 4 CIBER Epidemiology and Public Health CIBERESP 28029 Madrid Spain; 5 Cardiac Surgery Department, Agostino Gemelli University Policlinic 00168 Rome Italy; 6 Department of Extra Corporeal Circulation, UZ Gasthuisberg, KU Leuven 3000 Leuven Belgium

**Keywords:** Cardiopulmonary Bypass, Hematic antegrade repriming, Minimized extracorporeal circuits, Haemodilution, blood conservation, Enhanced recovery after surgery

## Abstract

*Background*: New era of cardiac surgery aims to provide an enhanced postoperative recovery through the implementation of every step of the process. Thus, perfusion strategy should adopt evidence-based measures to reduce the impact of cardiopulmonary bypass (CPB). Hematic Antegrade Repriming (HAR) provides a standardized procedure combining several measures to reduce haemodilutional priming to 300 mL. Once the safety of the procedure in terms of embolic release has been proven, the evaluation of its beneficial effects in terms of transfusion and ICU stay should be assessed to determine if could be considered for inclusion in Enhanced Recovery After Cardiac Surgery (ERACS) programs. *Methods*: Two retrospective and non-randomized cohorts of high-risk patients, with similar characteristics, were assessed with a propensity score matching model. The treatment group (HG) (*n* = 225) received the HAR. A historical cohort, exposed to conventional priming with 1350 mL of crystalloid confirmed the control group (CG) (*n* = 210). *Results*: Exposure to any transfusion was lower in treated (66.75% vs. 6.88%, *p* < 0.01). Prolonged mechanical ventilation (>10 h) (26.51% vs. 12.62%; *p* < 0.01) and extended ICU stay (>2 d) (47.47% vs. 31.19%; *p* < 0.01) were fewer for treated. HAR did not increase early morbidity and mortality. Related savings varied from 581 to 2741.94 $/patient, depending on if direct or global expenses were considered. *Discussion*: By reducing the gaseous and crystalloid emboli during CPB initiation, HAR seems to have a beneficial impact on recovery and reduces the overall transfusion until discharge, leading to significant cost savings per process. Due to the preliminary and retrospective nature of the research and its limitations, our findings should be validated by future prospective and randomized studies.

## Introduction

The new era of cardiac surgery has changed the paradigm of obtaining an acceptable survival rate to the adoption of Enhanced Recovery After Cardiac Surgery (ERACS) programs to reinforce every step of the perioperative procedure to augment the efficiency of cardiac surgery processes. Thus, the recommended intraoperative interventions are mainly oriented to maintaining temperature, glycemia, and oxygenation within a physiological range [1–3].

Due to the relevant contribution of the cardiopulmonary bypass (CPB) in this regard, the perfusionist approach in ERACS should be focused on the adoption of evidence-proven strategies to reduce the impact of CPB in terms of homeostasis, blood product requirements (BPR), and inflammatory response.

Minimally invasive extracorporeal circuits (MiECC) and repriming techniques like retrograde autologous priming (RAP) have been strongly recommended for blood-saving [1, 4, 5]. However, methodological weakness in current studies and heterogeneity between authors in the application of both is a matter of concern [6–11] about its beneficial effect in terms of the whole process.

Thus, with these concerns in mind, in 2014 our team developed the concept of Haematic Antegrade Repriming (HAR) as a standardized methodology, combining the benefits of MiECC and a new antegrade repriming method, improved with the application of vacuum-assisted venous drainage and the initiation of CPB with an empty venous line, to minimize the haemodilutional impact to 300 mL [12].

**Figure 1:**
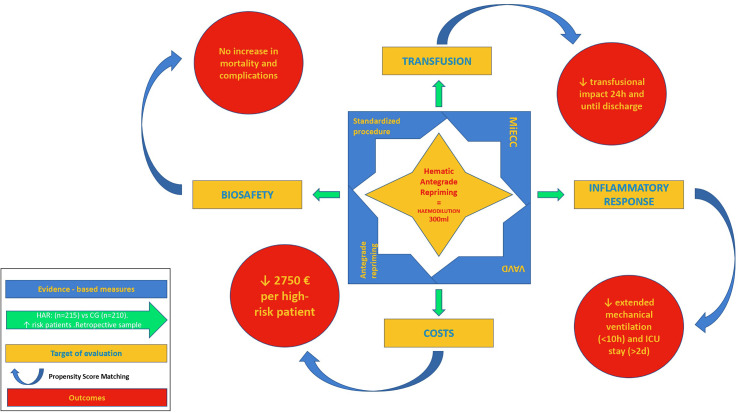
*Graphical abstract.* This figure summarizes the preliminary assessment of HAR’s potential benefits, to enhance postoperative recovery after cardiopulmonary bypass, considering a sample composed of high-risk patients and a propensity score matching methodology for the estimation of the effects. Abbreviations: MiECC – minimized extracorporeal circuit; VAVD – vacuum-assisted venous drainage; <10 h – lower than ten hours; <2 d – lower than two days.

Once proven the biosafety of the procedure in terms of gaseous microemboli (GME), and the beneficial effect of reducing the total embolic load delivered during CPB, seems mandatory to evaluate the potential benefit of HAR as a perfusion tool for ERACS (Figure 1).

## Material and methods

### Study design

Two retrospective cohorts of high-risk patients were compared by applying a propensity score matching (PSM), to estimate the effect of HAR, after the approval of the institutional ethics committee (IRB/EC Nr. 2019-10-3-HCUVA). Because of the retrospective nature of the research, no reported consent was required.

### Sample composition

**Figure 2:**
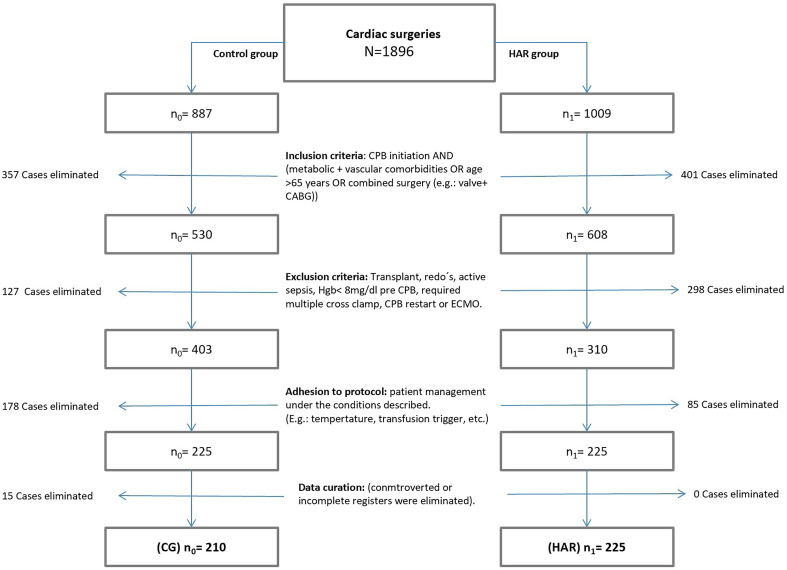
*Diagram of sample composition.* HAR group was recruited from 01/2014 to 12/2016, while CG was recruited from 01/2012 to 12/2013. Abbreviations: CG – control group; Hgb – Haemoglobin; CPB – cardiopulmonary bypass; ECMO – extracorporeal membrane oxygenation.

One thousand eight hundred and ninety-six patients undergoing elective cardiac surgery under CPB for four consecutive years in our institution were considered. After the application of exclusion criteria, 425 patients were assessed in a PSM model. The treatment group (HG) was recruited since the HAR procedure was standardized for every patient in our hospital, while the control group (CG) was composed, considering patients from a historical cohort, presenting similar preoperative conditions and treated under the same predefined clinical criteria to reduce the influence of biases (Figure 2).

### Inclusion/exclusion criteria

For election, patients required a preoperative logistic EUROSCORE index ≥5 [13] to be included and none of the exclusion criteria: preoperative anemia (Hgb <8 g/dL), emergent/urgent surgery, heart transplantation, endocarditis, preoperative renal failure, redo surgery within the first 48 h or multiple CPB initiations in a surgical process. Each case with controversial registers about clinical treatment, transfusion threshold, or postoperative management was also excluded from the study (Figure 2).

### Equipment

CPB was conducted with a Stöckert S5^®^ (Livanova™ PLC, London) heart-lung machine and the support of an AutoLog^®^ cell saver (Medtronic™ PLC, Minneapolis, Minnesota). Both groups received a biocompatible circuits including a Revolution^®^ centrifugal pump (Livanova™ PLC, London). Patients with a body surface area ≥1,8 m^2^ were oxygenated with a Capiox FX25^®^, included in a circuit treated with XCoating^®^ (Terumo Corp., Tokyo, Japan), while in smaller patients (<1,8 m^2^), a circuit with P.H.I.S.I.O.^®^ coating and the Inspire 6F^®^ oxygenator (Livanova™ PLC, London) was used.

### Procedure

CG patients underwent CPB, being connected to a conventional open circuit with ½ inch return line, (1350 mL of dynamic priming).

**Figure 3:**
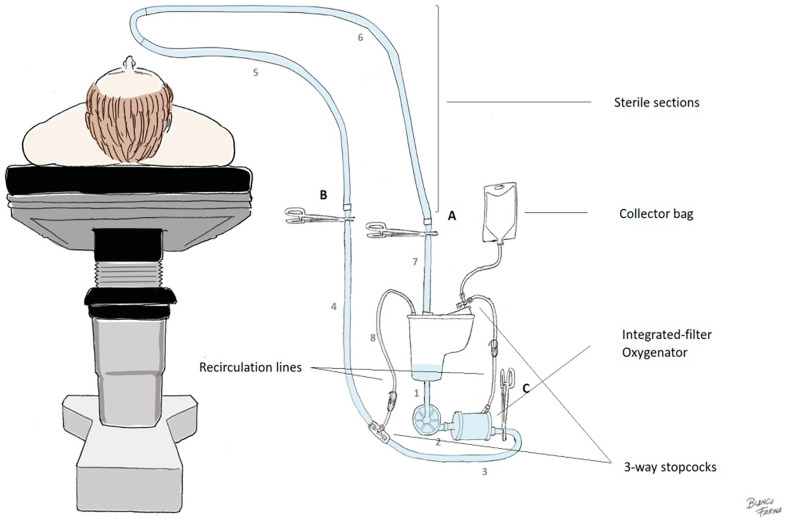
*Minimized extracorporeal circuit applied during haematic antegrade repriming.* Lines (dimensions): 1 – Post-reservoir line (3/8 x 35 cm); 2 – Pre-oxygenator line (3/8 × 40 cm); 3 – proximal arterial line (3/8 × 25 cm); 4 – medial arterial line (3/8 × 55 cm); 5 – distal arterial line (sterile) (3/8 × 135 cm); 6 – distal venous line (sterile) (3/8 × 105 cm); 7 – proximal venous line (3/8 × 40 cm); 8 – arterial recirculation line (clamp included) 1/8 (double male Luer-lock × 70 cm).

**Figure 4:**
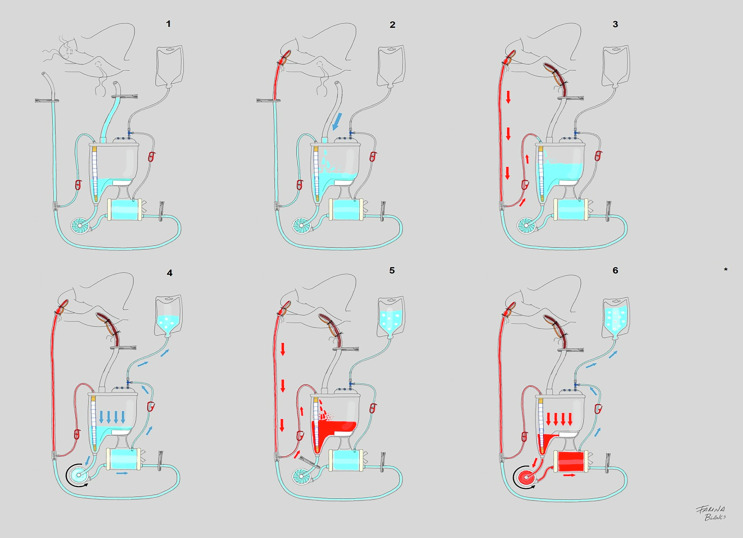
*HAR, the 6 steps procedure*. Step 1: The circuit is primed with 1000 mL of a balanced crystalloid solution. Then, venous and arterial lines are clamped. Step 2: Venous line content is drained to the reservoir by activating vacuum-assisted venous drainage (VAVD) and removing the venous clamp. Step 3: removing the arterial line clamp that is proximal to the patient and opening the arterial line recirculation, autologous blood discurs retrogradely pushing the crystalloid priming to the reservoir. Then, the arterial recirculation line clamp is closed to avoid blood mixing in the reservoir. Step 4: By opening the recirculation line of the oxygenator and setting the centrifugal pump (CP) to 2000 rpm, crystalloid priming is discarded into the collector bag until zero level in the reservoir is reached. Step 5: A clamp is placed after the reservoir and arterial line recirculation is opened again. Thus, retrogradely, 300 mL of arterial blood is sequestered in the reservoir (100–200 mL/min). Step 6: Setting CP to 2000 rpm and opening the recirculation line of the oxygenator and removing the clamp after the reservoir, CP and oxygenator are reprimed with autologous blood, displacing the priming and GME to the collector bag reducing haemodilution to only 300 mL. *CPB is initiated with VAVD activation once the venous return is obtained. Adapted from: [17].

HG patients received the standardized HAR circuit (760 mL) (Figure 3), being initially primed with 1000 mL of Isofundin^®^ (B. Braun, Melsungen, Germany). Then, the HAR procedure was applied by following a simple six-step procedure which does not increase the operative time. After recirculation, the circuit was clamped and offered to the surgeon. Then, lines were cut and the crystalloid in the venous line was drained to the hard-shell reservoir. All exceeding volume was diverted through the recirculation line of the oxygenator to a collector bag during the arterial cannulation. When the arterial line was connected priming was also discarded. Afterwards, 400 mL of autologous blood was retrogradely “sequestered” from the aorta during venous cannulation, to permit the succeeding antegrade repriming of the pump head and oxygenator. In order to avoid hypotension (MAP < 60 mmHg), backflow during sequestration did not exceed −250 mL/min and the anaesthesiologist considered the administration of phenylephrine boluses (0,01 mg) in some cases. Thereby, the haemodilutional impact of CPB initiation for treated patients represented 300 mL (Figure 4).

### Clinical management

All recruited patients were induced into general anesthesia with Propofol, Rocuronium, and Remifentanil in boluses. Hypnosis was maintained with sevoflurane according to monitoring, to avoid a bispectral index <60 (BIS, Medtronic, Minneapolis, USA). Ten mg/kg of tranexamic acid was administered during the induction, and a continuous infusion of 1 mg/kg/h was maintained during CPB. A 300 UI/kg bolus of sodium heparin was used as an initial dose to achieve anticoagulation. Target-activated clotting time (ACT) during CPB was > 440 s (Hemochron Signature Elite, Werfen, Spain), Heparin was reversed with protamine 1:1 after CPB. Afterward, the additional procoagulant strategy was thromboelastometric-guided (ROTEM Delta, Werfen, Spain) in every case to reduce postoperative coagulopathy.

Hgb drop below 8 mg/dL was strictly avoided. Therefore, the Renaflo II HF2000 hemofilter (Cantel Medical Corp, Little Falls, NJ, USA) was applied for continuous ultrafiltration (CUF) to compensate for hemodilution, when possible. Otherwise, if the circulating volume was not sufficient, red-blood-cells (RBC) transfusion was initiated [14].

During CPB, pump index and mean arterial pressure was maintained within ranges of 2–2.4 L/min/m^2^ and 50–80 mmHg, respectively. Cardioplegia was partially haematic (4:1), cold, intermittent, and predominantly antegrade for both groups and did not exceed 1000 mL of the crystalloid fraction. The temperature was actively maintained over 35 °C to avoid hypothermia [15, 16]. Following an alpha-stat strategy, euglycemia, osmolarity, and acid-base balance were also preserved in a physiologic range. After cross-clamp release, 3 mg of magnesium sulfate was administered, aiming to reduce the incidence of fibrillation.

### Statistical analysis

Baseline characteristics were described using means and standard deviations for continuous variables, as well as proportions for dichotomous ones, being compared with Student’s T-test and Pearson’s Chi^2^ test, respectively.

To estimate the effect of treatment on the outcome variates, a PSM (with kernel matching) model was applied [17]. Thus, a logistic regression model was used to estimate the propensity score to be treated with the HAR technique considering the covariates: sex, weight, age, body surface area, presence of diabetes mellitus, and type of surgery as well as the preoperative hematocrit, left ventricular ejection fraction, stroke and logistic EUROSCORE. Then, the balance between groups was assessed. A covariate was considered as balanced if the standardized difference was <10% and the variance rate was within the range of 0.5 and 2 points [18, 19]. Afterward, the average treatment effect on treated (ATT) was estimated by applying the bootstrap method (with 100, 1000, and 2000 iterations to verify the convergence of the model) to obtain 95% confidence intervals [17–19]. Then, the ATT estimation, regarding transfusion and ICU stay, was considered to calculate the economic impact of the HAR methodology. Statistical analyses were performed using Stata v.14, StataCorp LP.

## Results

Two hundred and twenty-five patients treated with HAR were compared to 210 controls. No significant differences were found between groups in terms of preoperative risk (Euroscore Log.: CG = 7.5%, HG = 6.8%, *p* = 0.42). Despite the differences in sex distribution (Sex (male): GC = 59.5% vs. HG = 71.1%; *p* = 0.01), no other significant differences were found in terms of weight, body surface area, preoperative Hb, comorbidities, or type of surgery (Table 1).

**Table 1:** Description of the covariate analysis.

Variate	CG			HG			
	*n*	Mean	SD	*n*	Mean	SD	*p-*Value
BSA	210	1.81	0.18	225	1.8	0.2	0.20
Weight	210	76.4	13.08	224	77.3	14.6	0.48
PrevHct	210	36.4	5.59	225	35.8	5.2	0.25
Age	210	65.7	11.18	225	64.2	12.7	0.18
LVEF	200	59.2	10.41	221	58	10	0.19
EUROSCORE I	205	7.5	10	221	6.8	8.6	0.42
PCreat	141	1.1	0.5	147	1.2	0.5	0.08
Sex	210	59.50%		225	71.1%		0.01*
DM	207	41.1%		223	38.6%		0.59
Pstroke	207	13%		223	7.6%		0.06
Surgery: Val	210	59.1%		225	55.1%		0.4
Surgery: CABG	210	20.9%		225	22.2%		0.75
Surgery: Mix	210	6.2%		225	8.9%		0.28
Surgery: Ao	210	9.1%		225	7.6%		0.57
Surgery: other	210	6.6%		225	6.6%		0.9

Abbreviations: Ao: Aortic; BSA: Body Surface Area; CABG: Coronary Artery Bypass Graft; DM: Diabetes Mellitus; ICU: Intensive Care Unit; LVEF: Left Ventricular Ejection Fraction; Val: Valvular.

Inconsistent data between different records were considered missing. *p-value < 0.05.

Regarding the PSM model, covariates appeared to be properly balanced (Table 2). ATT indicated that the treatment group’s exposure to any blood product until discharge was lower (GlobalBP: CG = 66.75% vs. GH = 6.88%, *p* < 0.001). Within the first 24 h, the exposure to RBC (RBC24: CG = 52.60% vs. HG = 5.05%, *p* < 0.001), plasma (FP24: CG = 11.22% vs. HG = 0.92%, *p* < 0.001) and platelets (PT24: CG = 32.07% vs. HG = 3.21%, *p* < 0.001), was significantly lower for treated. In the period within the first 24 h and hospital discharge, requirements of RBC (RBC > 24: CG = 38.55% vs. HG = 4.59%, *p* < 0.001) and plasma (FP > 24: CG = 4.45% vs. HG = 0.46%, *p* < 0.05) was also found to be lower for patients receiving HAR (Table 3).

**Table 2:** Balancing of preoperative variables.

Variate	HG	CG	Std dif. (%)	Variance rate
Sex (male)	0.71	0.70	1.7	0.99
Weight	77.61	76.67	6.9	1.28
Age	64.17	63.73	3.7	1.08
BSA	1.85	1.84	6.5	1.15
PrevHct	35.77	35.57	3.8	0.81
LVEF	57.90	57.88	0.2	1.18
DM	0.39	0.40	−1.8	0.95
PStroke	0.07	0.07	1.7	0.66
Surgery: Val	0.55	0.52	6.3	1.03
Surgery: CABG	0.23	0.27	−9.6	0.98
Surgery: Mix	0.09	0.08	2.5	0.85
Surgery: Ao	0.07	0.06	5.2	1.26
Surgery: Other	0.06	0.07	−1.1	0.9
EUROSCORE log.	2.85	2.85	0.6	0.95
PrevCrea	1.18	1.14	7.2	0.65

Abbreviations: Ao: Aortic; BSA: Body Surface Area; PrevHct: preoperative hematocrit; LVEF: Left Ventricular Ejection Fraction; DM: Diabetes Mellitus; PStroke: preoperative stroke; Val: valvular; CABG: Coronary Artery Bypass Graft; Mix: combined surgery; Ao: aorta; PrevCrea: preoperative creatinine. No significant differences were found (lowest *p*-value was Surgery: CABG = 0.33).

**Table 3:** Average treatment effect on treated (percentual estimation).

Variate	HG Mean	CG Mean	ATT	95% CI		*p*-Value
MecV > 10	12.62	26.51	−13.89	−23.68	−4.10	0.01*
ICU>2	31.19	47.47	−16.28	−27.13	−5.43	0.00*
Pulm Comp	5.50	8.70	−3.20	−9.43	3.03	0.31
Card Comp	0.92	1.59	−0.68	−3.14	1.79	0.59
Neur Comp	5.96	10.34	−4.37	−11.39	2.65	0.18
Glob Comp	1.38	5.68	−4.31	−9.03	0.41	0.07
Bleeding > 400	48.94	57.64	−8.70	−20.45	3.05	0.15
Exitus	5.50	12.71	−7.21	−14.61	0.19	0.06
RBC24	5.05	52.60	−47.55	−57.27	−37.83	0.00*
RBC > 24	4.59	38.55	−33.96	−43.08	−24.84	0.00*
FP24	0.92	11.22	−10.31	−15.75	−4.87	0.00*
FP > 24	0.46	4.45	−3.99	−7.76	−0.23	0.04*
PT24	3.21	32.07	−28.86	−37.48	−20.23	0.00*
PT > 24	0.92	4.93	−4.01	−8.37	0.35	0.07
GlobalBP	6.88	66.75	−59.87	−69.27	−50.47	0.00*

Abbreviations: MecV > 10 = Mechanical ventilation longer than 10 h; ICU > 2 = Intensive care unit stay longer than 2 days; Pulm. Comp. = Pulmonary complication; Card. Comp. = Cardiologic complications; Neur. Comp. = Neurologic complications; Glob. Comp. = Multiorganic failure; Bleeding > 400 = Drainage blood loss  > 400 mL within the first 24 hours; Exitus = survival; RBC24 = Red Blood Cell Transfusion the Day of Surgery; RBC > 24 = Red Blood Cell Transfusion after the Day of Surgery to Discharge; FP > 24 = Frozen plasma Transfusion on the Day of Surgery; FP > 24 = Frozen Plasma Transfusion after the Day of Surgery to Discharge; PT24 = Platelets Cell Transfusion on the Day of Surgery; PT > 24 = Platelets Transfusion after Day of Surgery to Discharge, GlobalBP: Exposure to any blood product until discharge.

Results correspond to 2000 iterations of bootstrap. Cases showed as *p*-value = 0.00 presented a *p-value < 0.05.

The requirement of mechanical ventilation after 10 h was significantly reduced for treated, (MV > 10 h: CG = 26.51% vs. HG = 12.62%, *p* = 0.005). Similarly, extension in ICU stay after 2 days was lowered in the treatment group (ICU > 2 d: CG = 47.47% vs. HG = 31.19%, *p* = 0.003) (Table 3). Otherwise, no significant differences were found between groups in terms of early mortality (CG 12.51% vs. HG 5.5%, *p* = 0.056), postoperative bleeding (Bleeding > 400: CG = 57.6% vs. HG = 48.9%, *p* = 0.14) and the incidence of postoperative complications (*p* > 0.05) (Table 3).

Considering the public unitary prices per blood component and per day of ICU stay in our region, the minimum cost saving related to the application of HAR was estimated as 581.7 $/patient (Table 4).

**Table 4:** Minimum direct cost estimation.

Unitary cost	Price ($)	Time	ATT	Cost/patient ($)
RBC transfusion*	230.59	24 hours	−47.55%	−109.64
		>24 h – discharge	−33.96%	−78.31
PT transfusion*	531.56	24 h	−28.86%	−153.51
FP transfusion*	201.72	24 h	−10.31%	−20.78
		>24 h – discharge	−3.99%	−8.04
Daily ICU stay	1299.33		−16.28%	−211.57
HAR application	0			0.00
Total amount				−581.70

Abbreviations: ATT: Average treatment effect on treated, RBC: Red Blood Cells; PT: platelets; FP: Fresh plasma; HAR: Hematic Antegrade Repriming; HCU: High Care Unit; ICU: Intensive Care Unit.

*Includes transfusional fix expenses. Prices obtained from: [15].

## Discussion

The transfusional effect estimated for HAR seemed to be superior to the isolated effect of MiECC, VAVD, RAP, and CPB initiation with an empty venous line. Albeit MiECC was recommended as I-A for blood conservation a decade ago, current evidence indicates that the overall effect in outcomes is controversial, due to not finding significant differences in terms of reoperation for bleeding, 30-day mortality, myocardial infarction, renal, and cerebral outcomes [6]. RAP nomenclature is also tainted by several limitations like the heterogeneity in practice and Vranken et al. warned about the high risk of biases behind the current level of recommendation related to the technique/s [8].

Regarding the use of VAVD, when applied in a range of suction not lower than −40 mmHg, seems to be a safe practice that contributes to reducing hemodilution and transfusion. The initiation of CPB with an empty venous line is a controverted perfusion strategy to reduce circulating volume and hemodilution, being required after the completion of HAR. Despite possible concerns about the emboli impact of any of both, there is recent evidence indicating that HAR represents a protective factor against emboli, reducing the embolic load delivered to the patient during CPB and the exposure to high embolic volumes [20]. Some authors pointed out that GME and sudden hemodilution play a major role in the aggression to endothelial glycocalyx, resulting in microvascular impairment, hypoperfusion, inflammatory response, and end-organ dysfunction [21–24].

However, current evidence supporting the individual benefits of the CPB initiation with an empty venous line or VAVD, in terms of enhanced recovery, is also poor [1, 25, 26]. Otherwise, our observations indicate that when all these techniques are combined in the standardized procedure described as HAR, priming hemodilution and BPR until hospital discharge are reduced until hospital discharge, representing a significant effect on the recovery. In terms of mechanical ventilation and ICU stay our study seems to be aligned with current evidence pointing out that exposure to sudden haemodilution during CPB is directly associated with the incidence of endothelial dysfunction, inflammatory response, and renal injury [22, 24, 27]. Some evidence indicates that MiECC application may reduce mechanical ventilation time, ICU length of stay, inflammatory response, cytokine release, and total stay [28–30].

Lower exposure to BPR and anemia are also related to lower postoperative morbidity and mortality [31–33]. However, our preliminary study only was able to observe that HAR applications did not represent significant differences in postoperative morbidity and bleeding, but a marked tendency to reduce in-hospital mortality by seven points was observed (*p* = 0.056). In this regard, we consider that the protective effect of the procedure could have been underestimated by the adoption of strict exclusion criteria, that eliminated redo cases, to improve the accuracy in measuring the effect on BPR.

Under an economic scope, HAR does not seem to represent any additional cost. Moreover, when applied to high-risk patients, just computing the direct cost of one unit when the exposure was avoided, the minimum unburdened savings exceeded 580 $/patient [34]. However, burdened costs of transfusion and ICU stay also include expenses like human resources, machinery, facility maintenance, transport, storage, side effects, and follow-up costs which should be considered [35]. Thereby, according to other authors [36–39], the saving per patient treated with HAR may reach the amount of 2741.94 $ (Table 5).

**Table 5:** Minimum global cost estimation.

Unitary cost*	Price ($)	Period	ATT	Cost/patient ($)
RBC transfusion	1183.22	24 h	−47.55%	−562.62
		>24 h to discharge	−33.96%	−401.82
PT transfusion	3457.06	24 h	−28.86%	−977.71
FP transfusion	1608.37	24 h	−10.31%	−165.82
		>24 h to discharge	−3.99%	−64.17
ICU >2d	3500		−16.28%	−569.80
HAR application	0			0
Total amount				−2741.94

Abbreviations: ATT: Average treatment effect on treated, RBC: red blood cells, PT: platelets, FP: fresh plasma, ICU > 2d: intensive care unit costs after the 2nd day of stay. *Overall cost per process was obtained from [36–39].

Several limitations should be considered in this preliminary study. Although the application of a PSM to estimate the causal effects of treatments can reduce the effect of biases when assessing retrospective samples, the results are affected by a higher model dependency than when a prospective randomized methodology is applied [40].

The exclusion criteria applied may have led to the underestimation of certain outcome variables. While the elimination of cases with clinical records compliant with criteria may have reduced the influence of biases, excluding all the patients requiring early reintervention due to active bleeding or cardiac tamponade may have attenuated the effect of HAR in all outcome variables.

To conclude, HAR seems to be a promising approach that could be included as a perfusion tool for ERACS programs, to promote the recovery of patients in terms of BPR, endothelial glycocalyx preservation, and embolic protection, without representing additional risks when applied under the specified methodology. However, future randomized and controlled studies are required to confirm our findings.

## Data Availability

Requestors wishing to access a de-identified minimal dataset for monitoring purposes of our published analyses, can apply to Juan Blanco-Morillo.
